# Co-downregulation of GRP78 and GRP94 Induces Apoptosis and Inhibits Migration in Prostate Cancer Cells

**DOI:** 10.1515/biol-2019-0043

**Published:** 2019-07-22

**Authors:** Tong Lu, Yue Wang, Kang Xu, Zhijun Zhou, Juan Gong, Yingang Zhang, Hua Gong, Qiang Dai, Jun Yang, Biao Xiong, Ze Song, Gang Yang

**Affiliations:** 1 Department of Urology, The First People's Hospital of Tianmen City, East No.1, Renmin Avenue, Tianmen City, Hubei 431700, P.R.China; 2 Sinopharm Wuhan Plasma-derived Biotherapies Co. Ltd., No.1 attached No.1, Zhengdian Gold Industrial Park Road, Jiangxia District, Wuhan, Hubei 430070, P.R.China

**Keywords:** GRP78, GRP94, prostate cancer, apoptosis, migration

## Abstract

**Background:**

Both glucose-regulated protein 78 kDa (GRP78) and glucose-regulated protein 94 kDa (GRP94) are important molecular chaperones that play critical roles in maintaining tumor survival and progression. This study investigated the effects in prostate cancer cells following the downregulation of GRP78 and GRP94.

**Methods:**

RNA interference was used to downregulate GRP78 and GRP94 expression in the prostate cancer cell line, PC-3. The effects on apoptosis and cell migration was examined along with expression of these related proteins.

**Results:**

Small interfering RNAs targeting GRP78 and GRP94 successfully down-regulated their expression. This resulted in the induction of apoptosis and inhibition of cell migration. Preliminary mechanistic studies indicated that caspase-9 (cleaved) and Bax expression levels were upregulated while Bcl-2 and vimentin expression levels were downregulated.

**Conclusion:**

Co-downregulation of GRP78 and GRP94 expression induces apoptosis and inhibits migration in prostate cancer cells.

## 1 Introduction

Prostate cancer (PCa) is the most common malignancy in middle-age and elderly populations. The increasing aging Chinese population has led to an increases in PCa incidence [1, 2]. Androgen receptors are known to play an important role in PCa development and progression. Currently, maximal androgen deprivation therapy (ADT) remains a common treatment of PCa[3]. However, PCa often develops resistance to ADT and can subsequently evolve into castration-resistant prostate cancer (CRPC) [4, 5]. Thus, there is a need to develop new therapies for CRPC. At present, gene therapy has opened new avenues for cancer treatment and has thus been attracting increasing attention. An important strategy in gene therapy is to interfere with both the transcription and translation processes of oncogenes and tumor suppressor genes to regulate their expression levels. Thus this regulation can affect tumor progression and improve patient prognoses [6, 7]. Therefore, identifying a genetic target suitable for CRPC therapy may lead to the development of novel therapeutics.

Glucose-regulated protein 78 kDa (GRP78) is a member of the heat shock protein family and is an important molecular chaperone. It functions by correcting protein folding errors and degrades irreversibly faulty polypeptides as part of the unfolded protein response (UPR). These functions help terminate or mitigate endoplasmic reticulum stress (ERS), in order to avoid apoptosis [8]. Glucose-regulated protein 94 kDa (GRP94) is another heat shock protein and also an important molecular chaperone. Both GRP94 and GRP78 play similar roles in the UPR process [8]. In addition, GRP94 is implicated in various signaling pathways; for example, this protein activates and sustains the MAPK and AKT/S6 signaling pathways in order to avoid apoptosis and promote cell proliferation [9, 10, 11]. Previous studies have shown that GRP78 and GRP94 are highly expressed in various tumor tissues and are involved in promoting tumor growth and invasion [12, 13, 14, 15, 16]. In this study, we used RNA interference technology to investigate the interaction between these two proteins in the PCa cell line, PC-3. We downregulated their expression levels both individually and simultaneously and examined the effects of this downregulation on biological behaviors (apoptosis, migration) as well as their possible mechanisms in PCa cells.

## 2 Materials and Methods

### 2.1 Cell culture

PC-3 cells (which were purchased from China Center for Type Culture Collection, Wuhan, China) were cultured in Dulbecco's Modified Eagle Medium (DMEM) containing 10% fetal bovine serum (FBS), streptomycin (100mg/ml) and penicillin (100 U/ml), in 5% CO_2_ at 37℃.

### 2.2 Immunohistochemical staining

Twenty PCa tissue cases were chosen for the experimental group, and 20 benign prostatic hyperplasia (BPH) tissue cases were used as a control group. Approval was obtained from all patients as well as from the ethics committee of the hospital prior to tissue collection for this experiment. The immunohistochemical staining process was as follows: tissue samples were fixed, paraffin-embedded and sectioned into 5 μm slices. The sections were dehydrated, dewaxed and then treated with hydrogen peroxide to block endogenous peroxidase activity. Antigen retrieval was performed by microwaving the sections, which were then blocked with 10% goat serum. Next, the sections were incubated overnight with anti-GRP78 and anti-GRP94 antibodies (Proteintech, USA). The sections were washed and then incubated with a secondary antibody from the ready-to-use SABC Staining Kit (Boster, China). Three fields were randomly selected under the microscope in each section, and the percentage of positively stained cells in the visual field was estimated. According to similar studies [17], the degree of staining was divided into five grades according to the proportion of positive cells: the proportion of positive cells <5% is negative (-), 5-25% is weakly positive (**±**), 26-50% is positive (+), 51-80% is moderately positive (2+), and =80% is strongly positive ( 3+), at the same time, pictures of these selected fields of view were taken to calculate their optical density (OD) values.

### 2.3 siRNA synthesis

All siRNAs, including those targeting GRP78 (siGRP78) and GRP94 (siGRP94) as well as a negative control siRNA (NC, non-targeted binding with any human gene sequence), were designed and synthesized by Invitrogen (USA). The sequences of the three RNA sets are shown in Table 1.

**Table 1:** Sequences of the three siRNA sets

siRNA	Sequence	Length
	S: AGUGUUGGAAGAUUCUGAUdTdT	19 bp
siGRP78	AS: dTdTUCACAACCUUCUAAGACUA	
	S: GAAGAAGCAUCUGAUUACCdTdT	19 bp
siGRP94	AS: dTdTCUUCUUCGUAGACUAAUGG	
NC siRNA	S: UUCUCCGAACGUGUCACGUdTdT	19 bp
	AS: dTdTAAGAGGCUUGCACAGUGCA	

### 2.4 Cell transfection

The reverse transfections were performed using the INTERFERin *in vitro* siRNA transfection reagent (Polyplus, France) according to manufacturer’s instructions. Specifically, siRNAs were diluted in 200 μl of Opti-MEM medium (Gibco, USA), combined with 12 μl of INTERFERin transfection reagent and incubated for 10 minutes at room temperature. Following incubation, the siRNA+INTERFERin transfection reagent mixture was transferred to a six-well plate containing 1.95 ml of FBS-free DMEM per well. PC-3 cells were incubated with the transfection mixture and cultured in a 37 ℃ incubator for 8 hours. Following this, 245 μl of FBS was added to each well, and the cells were returned to the incubator for further culturing. The final siRNA concentration in the NC, siGRP78 and siGRP94 groups was 50 nM. In the siGRP78+94 group, the final concentration was 50 nM siGRP78+50 nM siGRP94. No siRNA was added to the blank group.

### 2.5 Western blot analysis

48 hours after transfection, cells were harvested, lysed and centrifuged at 12,000g for 15 minutes to collect protein lysates. Protein concentrations were quantified, and equal amounts of protein were then separated by electrophoresis on a 12% SDS-PAGE gel and transferred to a PVDF membrane. The PVDF membranes were blocked with blocking solution and then incubated with a primary antibody overnight at 4℃. In this study, the following primary antibodies were used: anti-GRP78 and anti-GRP94 (Proteintech, USA); anti-caspase-9, anti-vimentin and anti-β-actin (Santa Cruz Biotechnology, USA); anti-Bcl-2, anti-Bax and anti-GAPDH (Abcam, UK). Following incubation with the primary antibodies, the membranes were incubated with an HRP-labeled secondary antibody (Santa Cruz) for 2 hours at room temperature. The BeyoECL Plus Kit (Beyotime, China) was used to visualize protein bands. The relative gray value of each band was determined using Quantity One 4.62 software (Bio-Rad, USA).

### 2.6 Apoptosis rate determination

48 hours after transfection, the cells were harvested and washed twice and resuspended in 500 μl binding buffer. Then, 5 μl PI and 5 μl FITC-labeled Annexin V (KeyGen, China) were added to the resuspended cell solution. This mixture was incubated for 20 minutes at room temperature in the dark. The apoptosis rates were determined by flow cytometry.

### 2.7 Determination of cell migration inhibition rates

Cell migration assays were performed using the Transwell system (Corning Life Sciences, USA). 48 hours after transfection, the cells were harvested and resuspended in serum-free DMEM. The cell densities were adjusted to 1×10 ^5^/ml, and 200 μl of cell suspension was added to each Transwell insert compartment. A total of 600 μl of medium containing 20% FBS was added to the lower compartment of each well of the 24-well plate, and the cells were cultured for 24 hours at 37 ℃. Then, the Transwell insert was removed and the cells on the outer surface of the Transwell insert were fixed, stained with crystal violet and photographed. To calculate the migration inhibition rate, the stained cells were lysed in 100 μl of 10% glacial acetic acid, and OD values were measured at 570 nm using a microplate reader. The migration inhibition rates were calculated with the following formula:

Migration inhibition rate = [1- OD value of experimental group / OD value of blank group] × 100%

### 2.8 Statistical methods

All data were statistically analyzed using SPSS 19.0 software (IBM, USA). Differences between three or more groups were assessed by one-way ANOVA. Differences between two groups were assessed using Student’s *t*-test. The data are expressed as the mean ± SD, and P<0.05 was regarded as statistically significant.

## 3 Results

### 3.1 GRP78 and GRP94 are highly expressed in PCa tissue

**Figure 1:**
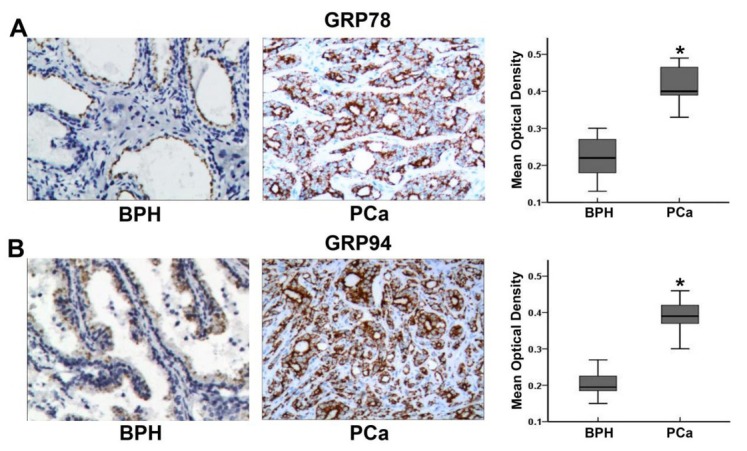
Expression of GRP78 and GRP94 in PCa and BPH tissues. A: Expression of GRP78 in BPH and PCa tissues, including a comparison of mean optical density values for GRP78 expression in each group. * indicates P<0.01 compared to BPH tissue. B: Expression of GRP94 in BPH and PCa tissues, including a comparison of mean optical density values for GRP94 expression in each group. * indicates P<0.01 compared to BPH tissue.

We first examined the expression of GRP78 and GRP94 in PCa tissue and benign prostatic BPH tissue via immunohistochemistry. Both proteins were mainly expressed in the cytoplasm and partially expressed in the cell membrane; positive staining was observed as brownish-yellow particles under the microscope. The results showed that GRP78 and GRP94 expression were moderately positive (2+) or strongly positive (3+) in 20 cases of PCa. GRP78 expression was strongly positive (3+) in 12 cases, while GRP94 expression was strongly positive (3+) in 14 cases. The expressions of GRP78 and GRP94 are shown in Figure 1. In all 20 BPH cases, GRP78 and GRP94 expression was either positive (+) or weakly positive (±). The mean OD value of the immunohistochemistry photographs was determined using HPIAS-1000 image analysis software (Champath Image, China). Analysis of the results demonstrated that the mean OD values for GRP78 and GRP94 expression in PCa tissue were significantly higher than those in BPH tissue.

### 3.2 siRNA-mediated downregulation of GRP78 or GRP94 affects protein expression in PCa cells

**Figure 2:**
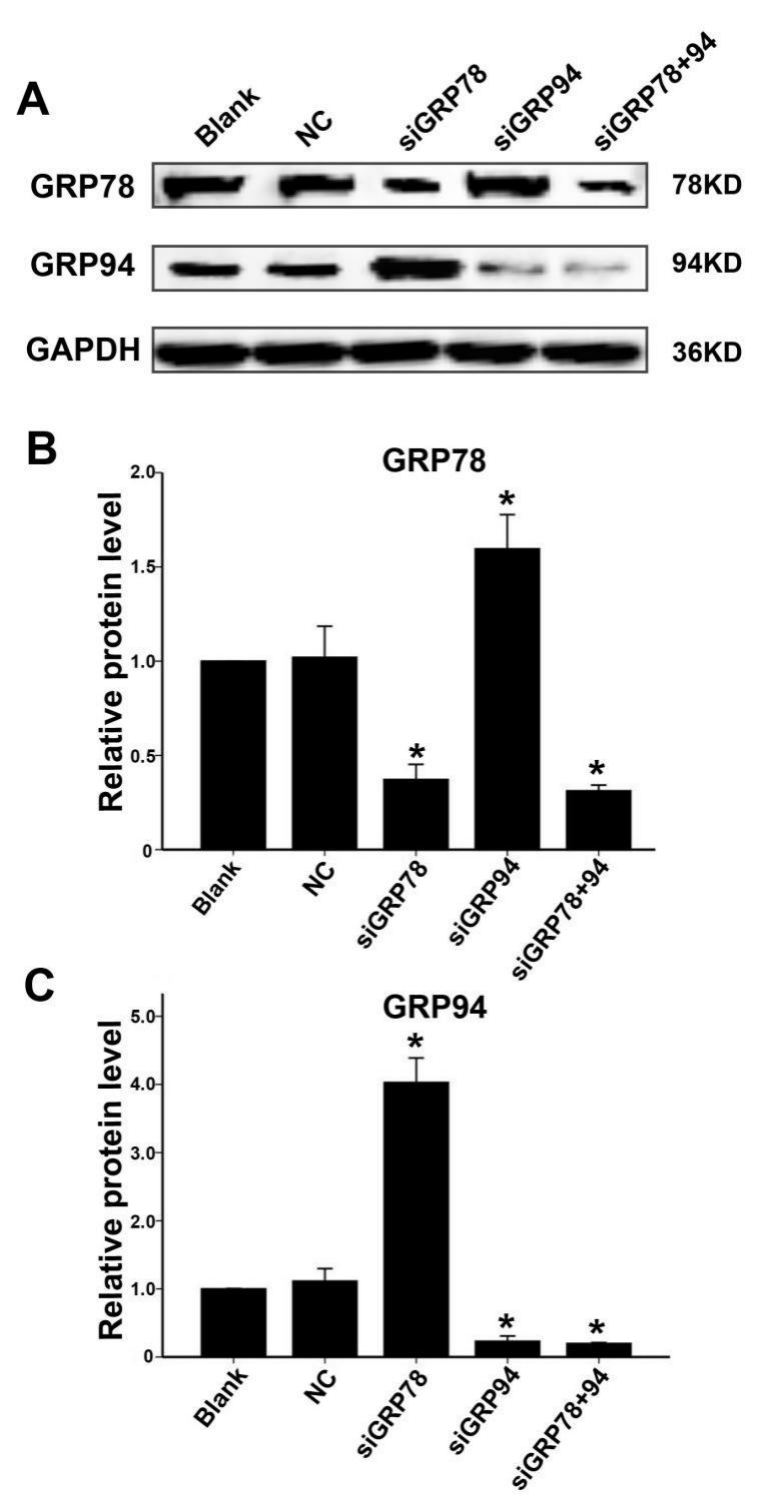
Changes in GRP78 and GRP94 protein expression in PC-3 cells transfected with siGRP78, siGRP94, or siGRP78 + siGRP94. A: GRP78 and GRP94 protein expression levels in the blank, NC, siGRP78, siGRP94 and siGRP78+94 PC-3 cell groups 48 hours after transfection. B：Relative GRP78 protein expression levels in all five groups (relative to the blank group). * indicates P<0.01 compared to the NC group. C: Relative GRP94 protein expression levels in all five groups (relative to the blank group). * indicates P<0.01 compared to the NC group

In this study, PC-3 cells were divided into the following groups: a blank group (blank; no siRNA added); a negative control group (NC; transfected with negative control siRNA); a GRP78 downregulated group (siGRP78; transfected with GRP78-targeting siRNA); a GRP94 downregulated group (siGRP94; transfected with GRP94-targeting siRNA); and a GRP78 and GRP94 co-downregulated group (siGRP78+94; transfected with both GRP94-targeting and GRP78-targeting siRNAs). Western blotting showed that in siGRP78-treated PC-3 cells, GRP78 expression was downregulated while there was a significant upregulation of GRP94 expression (P<0.01). Likewise, in the siGRP94 group, there was a decrease in expression of GRP94 expression and upregulation of GRP78 expression (P=0.001). In the co-downregulation siGRP78+94 group, the expression of both proteins was significantly downregulated. The protein expression levels of GRP78 and GRP94 in all five groups are shown in Figure 2.

### 3.3 Co-downregulation of GRP78 and GRP94 induces apoptosis in PCa cells

**Figure 3:**
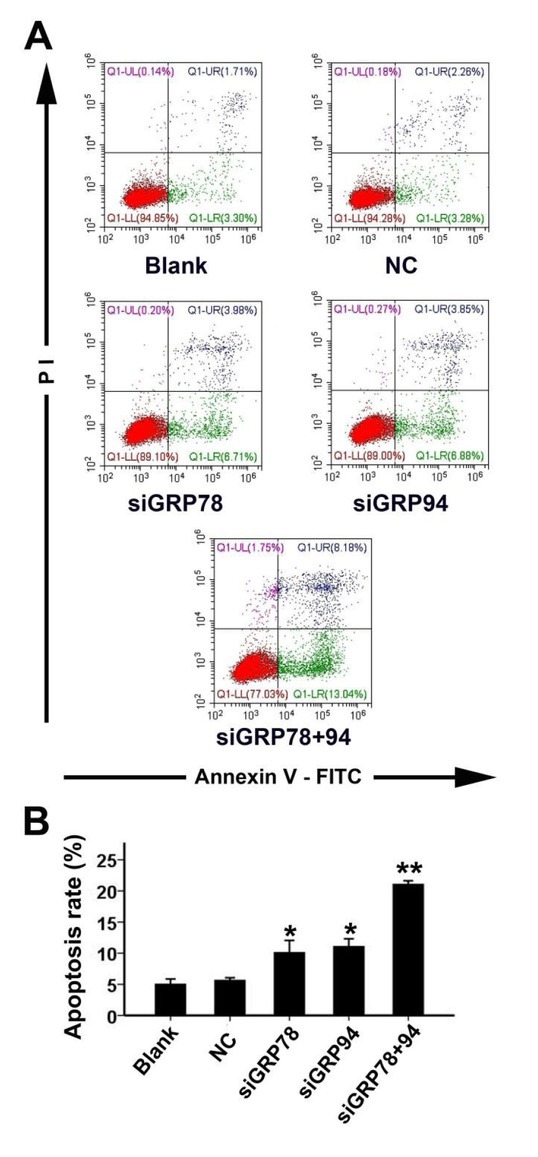
Apoptosis rates in PC-3 cells with co-downregulation of GRP78 and GRP94. A: 48 hours after transfection, the apoptosis rates in each group were measured using Annexin V-FITC + PI double staining. The UR （upper right quadrant）+ LR（lower right quadrant） area shows apoptotic cells. B: Comparison of apoptosis rates across all five groups. * indicates P<0.01 compared to the NC group; ** indicates P<0.01 compared to the siGRP78 and siGRP94 groups.

48 hours after transfection, we used Annexin V-FITC + PI staining to determine PC-3 cell apoptosis rates in each group by flow cytometry. As shown in Figure 3, the apoptosis rates in the siGRP78 and siGRP94 groups were significantly increased compared to the blank and NC groups. PC-3 cells cotransfected with siRNAs targeting GRP78 and GRP94, exhibited an apoptosis rate of approximately 20%, which is significantly higher than the rate observed in either the siGRP78 or the siGRP94 group.

### 3.4 Co-downregulation of GRP78 and GRP94 inhibits PCa cell migration

**Figure 4:**
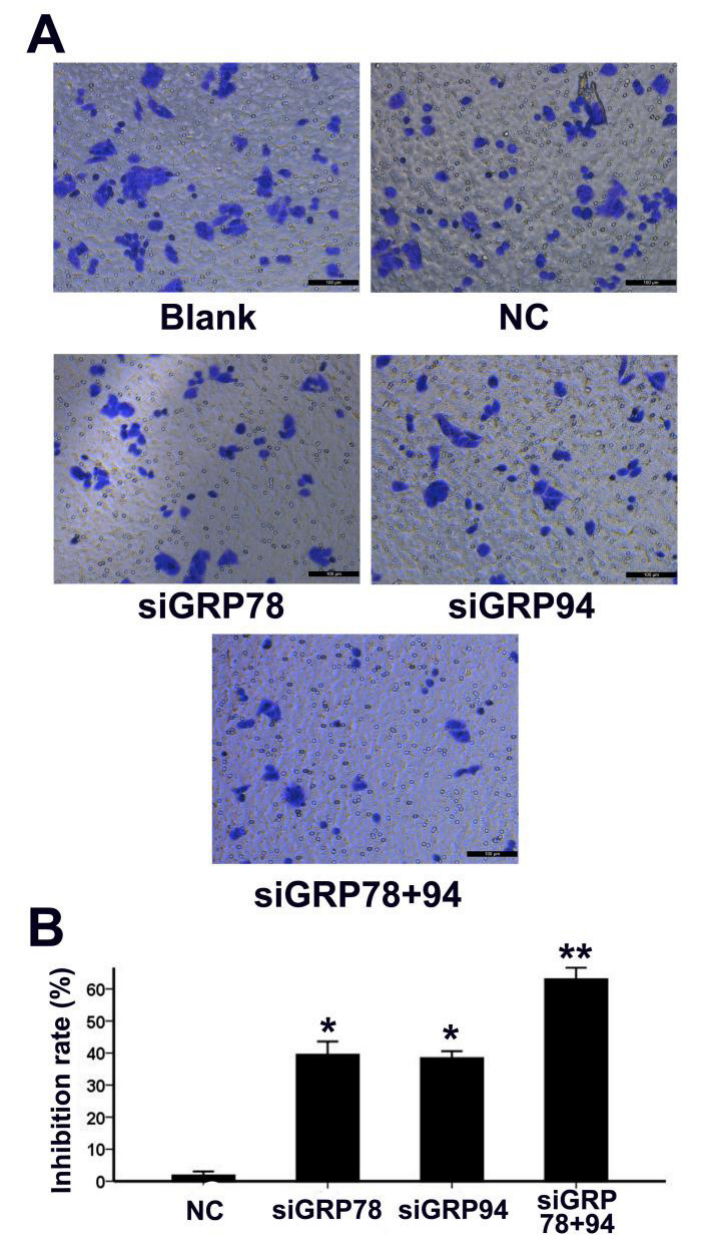
Co-downregulation of GRP78 and GRP94 inhibits PCa cell migration. A: The migratory ability of PC-3 cells in all five groups was determined using the Transwell system 48 hours post transfection. Photographs of crystal violet-stained cells that have migrated to the lower surface of the Transwell insert are shown. B: Comparison of migration inhibition rates across the NC, siGRP78, siGRP94 and siGRP78+94 groups. * indicates P<0.01 compared to the NC group; ** indicates P<0.01 compared to the siGRP78 and siGRP94 groups.

To determine the effect of GRP78 and GRP94 co-downregulation on PCa cell migration, we used the Transwell system and calculated migration inhibition rates in all five groups 48 hours after transfection. As shown in Figure 4, the migration inhibition rates in the siGRP78 and siGRP94 groups were significantly higher compared to the NC group. The migration inhibition rate in the siGRP78+94 group, was higher than in either the siGRP78 group or the siGRP94 group.

### 3.5 Effect of GRP78 and GRP94 co-downregulation on the expression of apoptosis- and migration-related proteins in PCa cells

**Figure 5:**
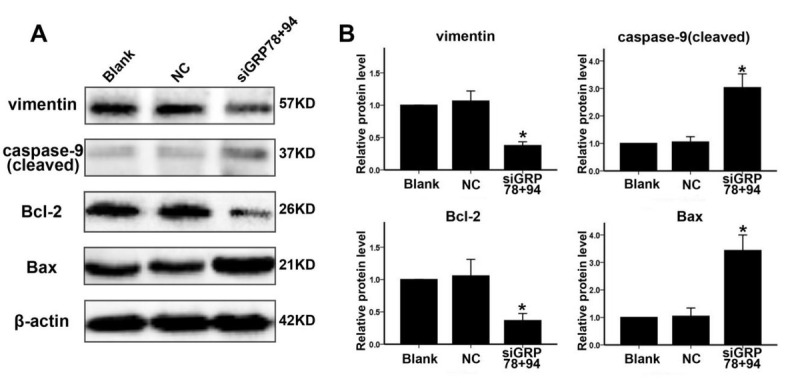
Co-downregulation of GRP78 and GRP94 affects the expression of apoptosis- and migration-related proteins in PCa cells. A: 48 hours after transfection, protein expression levels of caspase-9, Bcl-2, Bax and vimentin were evaluated in PC-3 cells in the blank, NC and siGRP78+94 groups. B: Relative expression levels of the four proteins examined in panel A. Protein expression was assessed in three experimental groups and was calculated relative to the blank group. * indicates P<0.01 compared to the NC group.

To further investigate the potential mechanism underlying GRP78 and GRP94 co-downregulation-induced apoptosis and inhibition of migration of PCa cells, we used Western blotting to examine protein expression levels of caspase-9, BCL-2, Bax and vimentin in PC-3 cells. The protein expression levels in this group were then compared to the protein expression levels in the blank and NC groups. As shown in Figure 5, in the siGRP78+94 group, the expression of the apoptosis-associated caspase-9 (cleaved) protein was upregulated. This group also displayed decreased expression of Bcl-2 and increased expression of Bax. In the siGRP78+94 group, the expression of vimentin, which is associated with cell migration, was also downregulated.

## 4 Discussion

PCa develops into castration-resistant prostate cancer (CRPC) after a period of androgen deprivation therapy (ADT), after which the efficacy of hormone therapy for prostatic tumors is significantly reduced. Gene therapy is attracting increasing attention as a novel form of CRPC treatment. Thus, it is critical to identify a gene target suitable for gene therapy-based PCa treatment.

GRP78 is a molecular chaperone protein that is mainly expressed in the endoplasmic reticulum (ER). This protein has multiple functions and includes aiding in protein folding and transport [18, 19]. It is also an important stress response protein and is involved in maintaining stable ER function under such conditions (e.g., low oxygen, low sugar and acidic environments). Thus, it plays important roles in cell survival, proliferation and migration [8]. Previous studies have shown that GRP78 is highly expressed in various tumor cells and is closely associated with tumor proliferation, metastasis and drug resistance [20, 21, 22]. Similarly, GRP94 is also mainly expressed in the ER, where it functions as a molecular chaperone protein. Though GRP94 displays complex physiological functions, it is also involved in numerous cellular functions which include assisting protein folding, transport, degradation, stabilizing cell states and ensuring cell survival during ER stress [8, 23, 24]. Studies have shown that GRP94 is highly expressed in various solid tumors and promotes tumor growth and metastasis [25, 26, 27]. Functionally, these two proteins are similar and possibly act synergistically in cancer cells.

In this study, we first examined the expression levels of these two proteins in BPH and PCa tissues. We found that both proteins were highly expressed, consistent with previous findings in various solid tumors [12, 13, 14, 15, 16, 20, 21, 22, 25, 26, 27]. This suggests that they may play an important role in PCa cells. Subsequently, we employed RNA interference technology to downregulate the expression of these two proteins in PCa cells. Our results demonstrated an inverse correlation between the two proteins. That is when GRP78 was downregulated, the expression of GRP94 was significantly upregulated. Likewise, when GRP94 was downregulated, the expression of GRP78 was upregulated. These results suggest the existence of a compensatory feedback mechanism between these two functionally similar proteins in PCa cells. When one protein is inhibited, the expression of the other increases in order to meet the functional demands of tumor cells in stressful environments (hypoxic, hypoglycemic and acidic environments) and to maintain cell proliferation and migration. However, the details of this compensatory feedback mechanism remains to be elucidated. Recent research has shown that the downregulation on the expression of either protein (GRP78 or GRP94) in some tumor cells, can to a degree, inhibit the proliferation or migration of tumor cells [28, 29, 30, 31]. However, the downregulation of both proteins at the same time in tumor cells has rarely been reported. To inhibit the compensatory effects of these two proteins, we introduced small interfering RNAs targeting both proteins (GRP78 and GRP94) in PCa cells. This resulted in significant decrease in expression levels of both GRP78 and GRP94.

To determine the effects of GRP78 and GRP94 co-downregulation on the biological behavior of PCa cells, we examined apoptosis rates and migratory abilities of PCa cells in each treatment group. We found that downregulation of GRP78 or GRP94 alone was able to induce apoptosis and inhibit the migration of PCa cells. However, when the two proteins were simultaneously downregulated, we found that there was a greater increase in apoptosis rates and significantly more inhibition of migration rates. This finding demonstrates that following the co-downregulation of both proteins, GRP78 and GRP94 are no longer able to functionally compensate for each other. As a result, this leads to a more pronounced inhibition of tumor cell characteristics. In light of these results, GRP78 and GRP94 may be promising targets in PCa therapy. However, further investigations involving more cell lines are necessary to comprehensively explore this mechanism. Furthermore, since differential basal levels of these molecular chaperones have been reported [32,33], GRP78/94-based treatments may only work with various efficacy.

Lastly, we investigated the potential mechanism underlying the co-downregulation-induced increase in apoptosis and inhibition of cell migration. Our results demonstrated that in the siGRP78+94 group, expression of the caspase-9 (cleaved) was upregulated. Additionally, Bcl-2 expression was downregulated while Bax expression was upregulated. Possibly the co-downregulation of GRP78 and GRP94 stimulates the mitochondrial apoptotic pathway by activating intracellular caspase-9 and increasing the Bax/Bcl-2 ratio [34, 35]. Vimentin expression was significantly decreased in the siGRP78+94 group. Vimentin is essential for cytoskeletal maintenance and as a result of the decrease in expression this may have an effect on inhibiting cell migration. Though the mechanism driving this observation still requires further investigation.

In summary, RNA interference technology can be effectively used to downregulate the expression of GRP78 and GRP94. This induces apoptosis and inhibits the migration of PCa cells. This siRNA-mediated co-downregulation has the potential to be used as a novel therapeutic method for PCa.
